# Supplementary material for: Oxidative Stress Response and E. coli Biofilm Formation under the Effect of Pristine and Modified Carbon Nanotubes
Source: Microorganisms. 2023 May 6;11(5):1221. doi: 10.3390/microorganisms11051221 (PMC10222281; doi:10.3390/microorganisms11051221)
Supplement: Supplementary file 1 [file microorganisms-11-01221-s001.zip › microorganisms-2342316-supplementary.pdf]

Supplementary Materials

Table S1. The effect of CNTs on the *soxS* expression: changes in the activity of β-galactosidase (OD420) in control, with PQ and CNTs

Time, min	SWCNTs	SWCNTs-COOH	SWCNTs-NH_2	SWCNTs-ODA	MWCNTs	MWCNTs-COOH	PQ	Control
**OD_{420}, the average value**								
0	0.041	0.056	0.052	0.045	0.043	0.020	0.039	0.039
30	0.056	0.067	0.055	0.055	0.052	0.055	0.094	0.054
60	0.077	0.087	0.084	0.081	0.077	0.077	0.157	0.076
90	0.124	0.134	0.133	0.131	0.118	0.094	0.233	0.114
120	0.156	0.176	0.150	0.163	0.136	0.125	0.285	0.145
150	0.183	0.193	0.185	0.191	0.153	0.149	0.334	0.171
180	0.205	0.206	0.197	0.212	0.172	0.175		0.184
210	0.231	0.233	0.235	0.239	0.192	0.202		0.215
240	0.241	0.254	0.231	0.251	0.195	0.214		0.225
270	0.265	0.271	0.257	0.270	0.231	0.231		0.247
**OD_{420}, the standard deviation**								
0	0.007	0.012	0.015	0.009	0.008	0.008	0.008	0.007
30	0.011	0.009	0.011	0.007	0.008	0.014	0.020	0.010
60	0.019	0.023	0.025	0.023	0.024	0.025	0.035	0.020
90	0.034	0.025	0.027	0.034	0.024	0.020	0.061	0.028
120	0.049	0.036	0.041	0.044	0.034	0.060	0.064	0.042
150	0.060	0.038	0.032	0.048	0.046	0.066	0.026	0.050
180	0.065	0.051	0.045	0.059	0.050	0.055	0.054	0.053
210	0.061	0.042	0.030	0.051	0.065	0.068	0.097	0.057
240	0.066	0.061	0.054	0.052	0.073	0.072	0.066	0.054
270	0.044	0.042	0.039	0.045	0.040	0.044	0.049	0.039
p								
	**$6.54{\times}10^{-4}$**	**$2.90{\times}10^{-6}$**	**$2.67{\times}10^{-4}$**	**$3.28{\times}10^{-4}$**	**0.025**	**0.015**		

Table S2. Antioxidant effect of CNTs in the presence of PQ

Time, min	SWCNTs + PQ	SWCNTs-COOH + PQ	SWCNTs-NH_2 + PQ	SWCNTs-ODA + PQ	MWCNTs + PQ	MWCNTs-COOH + PQ	PQ	Control
**OD_{420}, the average value**								
0	0.030	0.026	0.027	0.026	0.031	0.029	0.032	0.029
30	0.079	0.078	0.073	0.069	0.068	0.075	0.080	0.046
60	0.130	0.132	0.138	0.138	0.121	0.109	0.131	0.066
90	0.176	0.197	0.177	0.203	0.155	0.143	0.194	0.095
120	0.235	0.259	0.235	0.272	0.194	0.190	0.241	0.120
150	0.307	0.314	0.315	0.323	0.241	0.231	0.298	0.141

180	0.376	0.425	0.365	0.409	0.303	0.309	0.383	0.151
210	0.456	0.483	0.458	0.491	0.366	0.392	0.453	0.188
240	0.533	0.592	0.541	0.592	0.426	0.460	0.562	0.215
270	0.646	0.667	0.623	0.676	0.481	0.499	0.645	0.229
**OD_{420}, the standard deviation**								
0	0.004	0.008	0.004	0.004	0.004	0.006	0.013	0.004
30	0.010	0.018	0.023	0.009	0.005	0.011	0.010	0.010
60	0.009	0.010	0.023	0.005	0.010	0.023	0.017	0.009
90	0.020	0.003	0.033	0.011	0.007	0.019	0.016	0.020
120	0.047	0.021	0.097	0.011	0.039	0.016	0.024	0.047
150	0.037	0.058	0.004	0.054	0.023	0.039	0.041	0.037
180	0.052	0.021	0.032	0.015	0.067	0.063	0.045	0.052
210	0.059	0.025	0.057	0.022	0.042	0.058	0.032	0.059
240	0.004	0.076	0.013	0.048	0.079	0.077	0.043	0.004
270	0.057	0.139	0.013	0.092	0.058	0.007	0.020	0.057
p								
	0.170	**0.013**	0.141	**0.003**	**0.005**	**0.003**		

Table S3. ROS production under CNTs

Time, h	SWCNTs-COOH + *E. coli*	SWCNTs-NH_2 + *E. coli*	SWCNTs-ODA + *E. coli*	SWCNTs-COOH	SWCNTs-NH_2	SWCNTs-ODA	PQ	Control (*E. coli* K12)
**Units of fluorescence, the average value**								
0.5	140.85	21.30	13.15	128.88	32.75	10.00	25.43	21.63
1	195.52	37.74	24.02	133.38	50.22	11.38	62.18	38.00
2	285.36	130.44	61.63	175.75	113.75	14.75	212.05	86.56
3	388.32	299.80	127.98	217.88	187.50	20.50	468.50	160.03
4	504.35	578.30	227.21	269.00	289.75	28.00	836.53	238.53
5	634.42	859.00	344.10	335.38	412.25	35.75	1234.38	304.38
6	762.51	1243.49	508.56	384.38	559.63	47.50	1662.00	369.19
7	886.76	1600.89	601.77	454.75	719.25	60.50	2061.78	432.00
8	1071.48	1945.81	730.48	522.50	907.63	77.00	2481.05	501.91
9	1256.00	2312.58	850.66	575.25	1109.25	97.38	2873.13	570.84
**Units of fluorescence, the standard deviation**								
0.5	23.73	11.50	1.26	11.84	22.92	1.31	3.27	5.32
1	30.96	14.82	1.63	18.85	35.70	0.92	7.66	11.18
2	57.51	66.86	6.12	34.86	76.05	0.89	28.28	8.00
3	87.12	163.86	17.67	55.86	122.36	1.77	65.35	17.84
4	132.10	322.21	35.88	78.21	183.72	2.73	117.12	31.42
5	181.50	488.45	57.17	97.44	257.13	4.46	167.50	44.25
6	229.46	712.94	85.37	126.96	344.44	7.52	222.06	55.43
7	280.17	936.46	99.70	162.47	428.43	9.06	275.44	67.65
8	358.13	1139.06	119.31	198.50	538.95	12.25	336.32	77.47
9	436.70	1362.88	137.79	228.72	644.96	14.09	393.08	86.58

**Units of fluorescence, standard error of the mean (n = 40-96)**								
0.5	2.53	1.17	0.13	4.19	8.66	0.46	0.52	0.84
1	3.30	1.51	0.17	6.67	12.62	0.32	1.21	1.77
2	6.13	6.82	0.62	12.33	26.89	0.31	4.47	1.41
3	9.29	16.72	1.80	19.75	43.26	0.63	10.33	3.15
4	14.08	32.89	3.66	27.65	64.95	0.96	18.52	5.55
5	19.35	49.85	5.83	34.45	90.91	1.58	26.48	7.82
6	24.46	72.76	8.71	44.89	121.78	2.66	35.11	9.80
7	29.87	95.58	10.18	57.44	151.47	3.20	43.55	11.96
8	38.18	116.25	12.18	70.18	190.55	4.33	53.18	13.70
9	46.55	139.10	14.06	80.86	228.03	4.98	62.15	15.31
p								
	**0.0003**	**0.013**	0.067	**0.0037**	**0.020**	**0.002**		

Table S4. Expression of the *rpoS* under the effect of MWCNTs-COOH and SWCNTs-COOH

Time, h	SWCNTs-COOH	MWCNTs-COOH	Control
**OD_{420}, the average value**			
0	0.098	0.086	0.084
0.5	0.136	0.116	0.104
1.5	0.234	0.185	0.177
2.5	0.293	0.266	0.227
3.5	0.345	0.319	0.232
4.5	0.366	0.310	0.323
5.5	0.455	0.388	0.336
6.5	0.455	0.451	0.420
7.5	0.547	0.511	0.486
8.5	0.606	0.535	0.445
9.5	0.672	0.520	0.461
**OD_{420}, the standard deviation**			
0	0.023	0.004	0.004
0.5	0.024	0.010	0.015
1.5	0.052	0.059	0.040
2.5	0.081	0.048	0.057
3.5	0.040	0.045	0.069
4.5	0.065	0.018	0.047
5.5	0.081	0.022	0.044
6.5	0.095	0.090	0.075
7.5	0.102	0.082	0.094
8.5	0.214	0.137	0.065
9.5	0.223	0.130	0.044
p			
	**0.001**	**0.005**	
